# Dengue Virus Serotype 3 Subtype III, Zhejiang Province, China

**DOI:** 10.3201/eid1702.100396

**Published:** 2011-02

**Authors:** Jimin Sun, Junfen Lin, Juying Yan, Weizhong Fan, Liang Lu, Huakun Lv, Juan Hou, Feng Ling, Tao Fu, Zhiping Chen, Liming Cong, Qiyong Liu, Yanjun Zhang, Chengliang Chai

**Affiliations:** Author affiliations: Zhejiang Provincial Center for Disease Control and Prevention, Hangzhou, People’s Republic of China (J. Sun, J. Lin, J. Yan, J. Hou, F. Ling, Z. Chen, L. Cong, Y. Zhang, C. Chai);; Yiwu Municipal Center for Disease Control and Prevention , Yiwu, People’s Republic of China (W. Fan, T. Fu);; State Key Laboratory for Infectious Disease Prevention and Control, Beijing, People’s Republic of China (Q. Liu)

**Keywords:** Outbreak, dengue virus serotype 3, dengue fever, viruses, China, letter

**To the Editor:** Beginning in July 2009, physicians in the city of Yiwu, Zhejiang Province, People’s Republic of China, noted an outbreak of illness characterized by rash, headache, subjective fever, itching, anorexia, and arthritis. We present the results of the investigation of this outbreak, which was caused by dengue virus (DENV) serotype 3 (DENV-3) subtype III.

DENV-3 subtype III has been continuously circulating in the Indian subcontinent since the 1960s. The virus was first isolated from East Africa in 1985 in Mozambique and subsequently in Kenya (1991) and Somalia (1993) (*1*,*2*). Although dengue has occurred frequently in southern China, including Guangdong, Guangxi, Hainan, Fujian, and Zhejiang Provinces and in Taiwan (*3*–*6*), to our knowledge, DENV-3 subtype III has not been reported in China.

Yiwu is in the center of Zhejiang Province, southeastern China. This investigation included the entire town of Yiwu and towns that are part of the larger town of Yiting where the outbreak took place. We reviewed medical records and conducted prospective surveillance at all hospitals, health centers, and outpatient clinics in Yiwu to identify patients with suspected dengue fever (DF) during July 1 through October 31, 2009. According to the diagnostic criteria for DF (WS216–2008) enacted by the Chinese Ministry of Health, a patient with suspected disease had at least 2 of the following symptoms: acute onset of rash, headache, subjective fever, itching, anorexia, or arthralgia. Patients with suspected disease were asked to provide blood specimens during the acute phase (within 7 days after symptom onset).

Serum samples were tested by ELISA for immunoglobulin (Ig) M against DENV by using the E-DEN01M kit (Panbio, Sinnamon Park, Queensland, Australia). Acute-phase serum samples were tested by real-time PCR for DENV RNA, according to the diagnostic criteria for dengue fever enacted by the Chinese Ministry of Health. Samples that were positive for DENV-3 by real-time PCR were inoculated into *Aedes albopictus* mosquito clone C6/36. Primers for reverse transcription–PCR and sequencing of the envelope gene of DENV isolates were used to identify DENV (*4*).

We considered a patient to have a confirmed case if DENV RNA was detected in the serum by real-time PCR or if IgM against DENV was present. A patient was considered to have a clinically diagnosed case if he or she had acute onset of rash, headache, subjective fever, itching, anorexia, and leukopenia, and lived in Qingsu, Fantianzhu, Xitian, or Shangzhai (4 adjoining villages in the area of the first confirmed case).

The sequences of isolates from case-patients were compared with published sequences by using the BLAST program (www.ncbi.nlm.nih.gov/BLAST/), and phylogenic analysis was calculated with PAUP 4.0 β 10 (*7*), which ran an unrooted tree with 1,000 bootstrap replicates.

We identified 196 cases of DF; 279 suspected cases were excluded, and no cases of dengue hemorrhagic fever or dengue shock syndrome were found. Of DF cases, 71 (36.2%) were confirmed and 125 (63.8%) were clinically diagnosed. Acute-phase serum samples were collected within 7 days after the onset of illness from 350 patients with suspected DF, and dengue virus RNA was detected in samples from 65 patients (18.6%). Six samples had IgM against DENV.

Twenty-six samples positive for DENV RNA by real-time PCR were randomly selected to isolate viruses; 23 isolates were cultured. All isolates were amplified by reverse transcription–PCR, and amplicons were sequenced. The envelope gene sequences of all isolates were identical and 1,479 nt in length. All sequences had 99% similarity to 1 DENV serotype 3 partial envelope gene (GenBank accession no. AM746229), which had been detected in Jeddah, Saudi Arabia, in 2004. According to evolutionary analysis (Figure), sequences of our study were also most closely related to the isolate from Saudi Arabia, which suggests that the outbreak may have been initiated by imported cases from the Indian subcontinent or western Asia.

**Figure:**
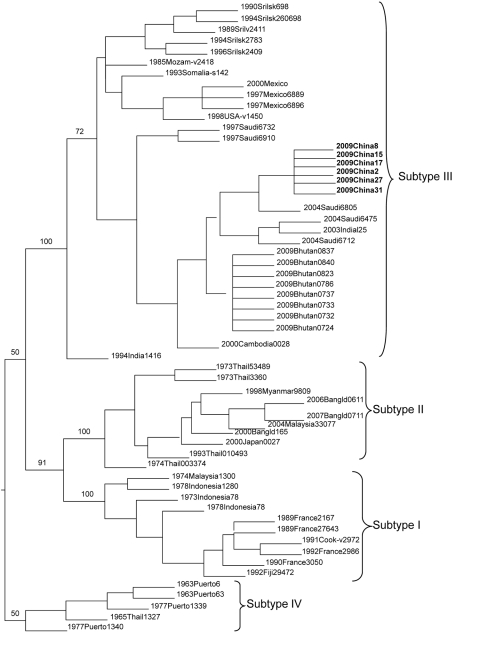
Evolutionary analysis of dengue virus isolates from this study (**boldface**) compared with established dengue virus serotype 3 subtypes, Zhejiang Province, People’s Republic of China, 2009.

The date of symptom onset among patients with confirmed or clinically diagnosed cases ranged from July 20 to October 4, 2009. Cases peaked in early September and subsided in early October. The median age of patients with confirmed or probable disease was 47.3 years (range 3–96 years). Infections occurred in all age groups, but most infections occurred among persons 41 to 65 years of age; 118 (60.2%) were women, and 172 were farm workers.

Confirmed and clinically diagnosed cases occurred in 18 villages, which were part of 7 towns. Most cases (182) were reported in Yiting, where the first case was confirmed, and in particular, were in persons who lived in the villages of Qingsu, 100 cases; Fantianzhu, 49 cases; Xitian, 19 cases; Shangzhai, 4 cases; and Xiateng, 4 cases.

The outbreak shows that DENV-3 subtype III is easily transmitted among humans and mosquitoes and can adapt efficiently to a new area. Other countries where the climate is similar to that of Zhejiang Province (subtropical monsoon) should be aware of the risk for expansion of DENV-3 subtype III transmission. Clinical vigilance and strong epidemiologic and laboratory surveillance are essential**.**

## References

[1] Gubler DJ, Sather GE, Kuno G, Cabral JR. Dengue 3 virus transmission in Africa. Am J Trop Med Hyg. 1986;35:1280–4. 3789276 10.4269/ajtmh.1986.35.1280

[2] Kanesa-thasan N, Chang GJ, Smoak BL, Magill A, Burrous MJ, Hoke CHJ. Molecular and epidemiologic analysis of dengue virus isolates from Somalia. Emerg Infect Dis. 1998;4:299–303. 10.3201/eid0402.980220 9621203 PMC2640142

[3] Qiu FX, Gubler DJ, Liu JC, Chen QQ. Dengue in China: a clinical review. Bull World Health Organ. 1993;71:349–59. 8324854 PMC2393493

[4] Juying Y, Yiyu L, Jingqing W, Haiyan M, Yan F, Wen SH, The etiological study of a dengue fever outbreak and the molecular characterization of the dengue virus isolates in Zhejiang Province. Chin J Virol. 2006;22:339–44.

[5] Lei L. Comparison of epidemiological characteristics of dengue fever between 2002 and 2006, Guangzhou. S China J Prev Med. 2008;34:18–21.

[6] Shaojian C, Rongtao H, Nengxiong Z, Jianming O, Yansheng Y. Epidemiological analysis of dengue fever outbreak in 2004 from Fujian Province. Haixia J Prev Med. 2006;12:32–4.

[7] Swofford DL. PAUP*: phylogenetic analysis using parsimony and other methods, Version 4.0b9. Sunderland (MA): Sinauer Associates; 2002.

